# Handgrip Strength and Vitamin D as Predictors of Liver Fibrosis and Malnutrition in Chronic Hepatitis C Patients

**DOI:** 10.1155/2021/6665893

**Published:** 2021-04-02

**Authors:** Sami A. Gabr, Ahmad H. Alghadir

**Affiliations:** ^1^Rehabilitation Research Chair, College of Applied Medical Sciences, King Saud University, Riyadh, Saudi Arabia; ^2^Department of Anatomy, Faculty of Medicine, Mansoura University, Egypt

## Abstract

**Background:**

In patients with chronic hepatitis C (CHC), a negative impact of associated malnutrition on both morbidity and mortality was reported. We aimed to elucidate the efficacy of serum liver fibrosis markers (fibronectin (FN), hydroxyproline (Hyp), and hyaluronic acid (HA)) and their respective indices (HA index, Hyp index, and FN index) and vitamin D status in predicting malnutrition associated with liver fibrosis in CHC patients and to investigate their association with the value of current clinical malnutrition assessment tools subjective global assessment (SGA), handgrip strength (HGS), and muscle mass scores (SGA, BMI, MAMC, and HGS).

**Materials and Methods:**

A cross-sectional study was conducted on 80 patients aged 40-60 years with proven viremia, HCV antibodies, HCV-RNA positivity, genotype determinations, and established chronic hepatitis C virus for more than 6 years and 80 control subjects. SGA, HGS, and muscle mass score (MAMC) were estimated in both patients and control subjects. Based on SGA scores, CHC patients were classified into three groups: well nourished (*n* = 12; SGA-A); mild or moderately malnourished (*n* = 25; SGA-B); and severely malnourished (*n* = 43; SGA-C). Liver fibrosis markers, inflammatory indicator *α*-Fetoprotein (AFP), tumor necrosis factor-alpha (TNF-*α*), 25-hydroxyvitamin D, and PTH were estimated using immunoassay techniques.

**Results:**

CHC patients with moderate and severe malnutrition SGA scores showed a significant decline in the levels of vitamin D, increased PTH, and lower values of HGS and muscle mass indices compared to well-nourished patients and control subjects. In addition, malnutrition, vitamin D deficiency, and lower values of HGS, MAC, TSF, and MAMC showed significant correlation with liver severity among CHC patients. Liver fibrosis markers Hyp, HA, FN, APRI, HypI, HAI, and FNI as noninvasive biomarkers showed significant correlation with both severity of liver diseases and associated malnutrition, especially in cirrhotic HCV patients (F4) compared to those with significant fibrosis (F2–F3).

**Conclusion:**

The results showed that deficiency in vitamin D levels, HGS, SGA, and muscle mass scores (MAC, MAMC, or TSF) could be used as markers of liver pathogenicity in patients with CHC. In addition, the study concluded that noninvasive biomarkers Hyp, HA, FN, APRI, HypI, HAI, and FNI separately or in association with vitamin D status, HGS, SGA, and muscle mass scores (MAC, MAMC, or TSF) were significantly associated with an incidence of malnutrition between ~70.5% and 89.6% of CHC patients with significant fibrosis and cirrhosis.

## 1. Introduction

In patients with hepatic viral infections (HCV and HBV), malnutrition was shown to be associated with morbidity and mortality particularly in patients with severe liver complications such as liver fibrosis, cirrhosis, cancer, and chronic viral hepatitis (CHC) [1, 2]. In patients with compensated diseases and liver cirrhosis, malnutrition was significantly associated with liver diseases in 20% to 60% of the patients [3]. In patients with HCV, malnutrition was found early in association with viral infections and proceeds progressively throughout the spectrum of HCV disease [4].

In patients with CHC, the rapid progression of liver damage was shown to be associated with malnutrition [5]. Frequently, CHC patients become malnourished since they are unable to meet their nutritional requirements [6].

In chronic liver disease, the pathogenesis of malnutrition is multifactorial and significantly includes a reduction in oral intake. Many consequences such as anorexia, nausea, dietary restrictions, altered nutrient biosynthesis, malabsorption, abnormalities of carbohydrate, lipid and protein metabolism, and a hypermetabolic state have significantly resulted from malnutrition [7].

In severe liver diseases, many effects of malnutrition like poor energetic intake due to a proinflammatory state and the presence of ascites along with lower levels of both calcium and magnesium were significantly provided in hepatic patients [3, 8]. Furthermore, nutrient digestion, absorption, storage, and metabolism were greatly affected in patients with chronic hepatic diseases, leading to deficiency in mineral and vitamin uptake and progressive protein-energy malnutrition [9]. Previous studies showed that deficiency in vitamin D levels along with disorder in the levels of parathyroid hormone (PTH) was significantly associated with liver fibrosis and components of hepatic dysfunction in patients with CHC [10], whereas the natural history and the treatment of CHC infection were associated with the uptake and the levels of 25-vitamin D in the serum [11]. It was reported that intake of vitamin D alone or combined with interferon-alpha significantly increases the innate and adaptive immune responses against HCV and suppresses HCV viral replication in Huh-7.5 cells [12]. The potential protective role of vitamin D proceeds via the expression of interferon-stimulated genes (ISGs) and a production of a synergistic effect with interferon-alpha.

Assessment of the degree of liver fibrosis correctly plays a pivotal role in controlling the progression of the disease and evaluating the prognosis for CHC infection as well as arranging for good new therapeutic strategies against CHC [13, 14].

Histological examination was identified to be the gold standard methodology for diagnosis, assessing of the degree of hepatic fibrosis, and estimating prognosis among hepatic diseases [15]; however, in most circumstances, the use of liver biopsy in diagnosis sometimes results in false positive and false negative diagnoses among severe hepatic cases [16]. Thus, noninvasive methods were required for accurate diagnoses of liver fibrosis and cirrhosis as well as to differentiate between the early and past stages of liver fibrosis [17]. Noninvasive reliable biomarkers were shown to be an active area for clinical interest in the diagnosis of CHC, grading hepatic fibrosis, and monitoring of outcome measures following the treatment of HCV-infected patients [18–20].

In recent studies, liver fibronectin, hydroxyproline, hyaluronic acid, liver function, and platelet counts and their respective indices like APRI, HA index, Hyp index, and FN index were significantly correlated with severity and liver fibrosis scores in different hepatic diseases particularly in CHC patients [18–21].

However, the correlation between these noninvasive biomarkers and their importance in predicting and evaluating malnutrition in CHC patients with severe hepatic dysfunction was not fully elucidated. Frequently, in patients with CHC, the relation between malnutrition and severity of liver diseases is rarely undiagnosed [4, 21]. In addition, there are no gold standard methods for the diagnosis of malnutrition in patients with CHC [22, 23]. Different parameters such as body mass index (BMI) and subjective global assessment (SGA) along with the handgrip strength (HGS) were previously applied to evaluate malnutrition associated with liver cirrhosis [24–27].

In the current study, we aimed to elucidate the efficacy of serum liver fibrosis markers (fibronectin, hydroxyproline, and hyaluronic acid) and their respective indices (HA index, Hyp index, and FN index) and vitamin D status in predicting malnutrition associated with liver fibrosis in CHC patients and to investigate their association with the value of current clinical malnutrition assessment tools (SGA, BMI, MAMC, and HGS).

## 2. Materials and Methods

### 2.1. Subjects

A total of 80 patients aged 40-60 years with established chronic hepatitis C virus for more than 6 years were recruited in this descriptive cross-sectional study. Patients with proven HCV antibodies, HCV-RNA positivity, and genotype determinations were included in this study.

In addition, age- and sex-matched 80 healthy subjects aged 40-60 years were selected as controls from a population undergoing a standard annual physical examination and biological measurements for medical insurance [19, 20]. The data were collected from December 2012 to May 2013 from the outpatient department of Gastroenterology Surgical Centre, Faculty of Medicine, Mansoura University, Mansoura, Egypt. Patients with other liver complications such as HBV, HIV, cirrhosis status, hepatocellular carcinoma, alcohol intake, drug abuse, chronic renal insufficiency, chronic pancreatitis, and cognitive alterations like hepatic encephalopathy were excluded from this study. Also, patients who received medications that affect VitD3 metabolism were also excluded. Based on the ethical guidelines of the 1975 Declaration of Helsinki, the study protocol was reviewed and approved by ethical committee of Rehabilitation Research Chair (RRC), King Saud University, Kingdom of Saudi Arabia, under file number ID RRC-2016-092. An assigned informed consent was obtained from all participants prior to data collection. A heparinized syringe was used to collect blood samples from all subjects, and plasma samples were obtained from whole blood following centrifugation for 1 min at 1400 rpm. The samples were kept frozen at 20°C until use. Demographic and clinical data of the participants are in Table 1.

### 2.2. Anthropometric Measurements

A standardized procedures such as a tape measure and calibrated Salter Electronic Scales (Digital Pearson Scale; ADAM Equipment Inc., Columbia, MD, USA) were used to estimate both height and weight of all participants. BMI and waist to height ratio (WHtR) as parameters of adiposity were also calculated according previously validated universal cutoff values [28]. The Slim Guide skinfold caliper was used to measure the triceps skinfold, and the mean of three measurements was taken. Furthermore, the midarm circumference (MAC) was measured at the midpoint between the tip of the acromion and the olecranon process on the nondominant side of the body with a flexible tape measure with the subject sat upright with their arm flexed at 90° [27, 29]. Midarm muscle circumference (MAMC) was calculated using the MAC and the TSF according to standard equations given as follows [30]: {MAMC = MAC (cm) − (0.314 × TSF (mm))} [27, 29].

### 2.3. Assessment of Liver Enzymes and HCV Markers

For all subjects, blood markers were performed on the day of biopsy or within 5 days after liver biopsy. Serum AST, ALT, total bilirubin, and albumin were performed using Max Discovery™ Color Endpoint Assay kits (Cat. No.BO-5605-01 and BO-3460-08, Bioo Scientific Co., USA). *α*-Fetoprotein (AFP) and tumor necrosis factor-alpha (TNF-*α*) were estimated in the serum of all participants by using a sandwich ELISA assay and immune assay kits (AFP; R&D Systems, USA; TNF-*α*; BD Biosciences, USA, respectively). In addition, HCV antibody (anti-HCV) and HCV-RNA were estimated in patients with chronic HCV by using third-generation enzyme immunoassay (EIA) (Axsym HCV 3.0, Abbott Laboratories, Chicago, IL) and an in-house direct reverse transcriptase-polymerase chain reaction (RT-PCR) assay, respectively. Also, HCV genotypes were identified by a reverse hybridization method using Line Probe assay (INNO-LiPA HCV II kit, Innogenetics, Zwijndrecht, Belgium). The data were interpreted according to the manufacturer's instructions.

### 2.4. Assessment of Liver Fibrosis Markers

An enzyme-linked immunosorbent assay (ELISA) was performed to estimate both serum hyaluronic acid (HA) (HA-binding protein; Corgenix kit) for HA and hydroxyproline by using immune assay kits (Hyp; Cat. No. E0621Hu; Uscn Life Science Inc., Wuhan) for Hyp, respectively. Tissue fibronectin (FN) concentrations were estimated using an immune assay ELISA kit (ABIN1874233, Atlanta, GA30338, USA) at a wavelength of 450 nm using a spectrophotometer. The concentration of fibronectin was then determined by comparing the OD of the samples to the standard curve as previously reported [19, 20].

In addition, to predict liver fibrosis, different indices were calculated. ALT and AST indices were calculated by dividing the patient's test results by the upper limit of normal (40 IU/L) for the test. The AST/platelet count ratio index (APRI) was calculated as AST index/platelet count divided by 10^3^ times 100. The HA, Hyp, and FN indices were calculated by dividing the patient's test results by platelet count ratio divided by 10^3^ times 100 [17, 19, 31, 32].

### 2.5. Assessment of Nutritional Scores

A prevalidated subjective global assessment (SGA) score with good to excellent interobserver reproducibility was used to calculate nutritional status in all subjects as previously reported [26, 33]. Based on the SGA score, CHC patients in this study were classified prospectively into three groups: well nourished (*n* = 12; SGA-A), mild or moderately malnourished (*n* = 25; SGA-B), and severely malnourished (*n* = 43; SGA-C) [33]. The control group reported a normal nutritional status (well nourished; SGA-A). Both SGA and body mass index (BMI) are used for the evaluation of malnutrition as previously reported [33].

### 2.6. Assessment of Handgrip Strength

Handgrip strength of both the right and left hands with 0.1 lbf accuracy was measured by using a manual hydraulic dynamometer label JAMAR (Hydraulic Hand Dynamometer® Model PC-5030 J1, Fred Sammons, Inc., Burr Ridge, IL, USA) [34, 35]. The measurements were performed in the standard position, and each participant was seated in a straight-backed chair. Then, he was asked to squeeze the dynamometer two times with each hand. For each hand, approximately 2 min rest lapsed between trials and control for the effects of fatigue on each hand alternated, and the best value of two attempts was recorded [35]. The inter-rater technical error of measurement was less than 2.5% for both hands. Based on grip strength measurements (HGS), patients' muscle strength was expressed as normal (HGS: ≥300 mmHg), moderate (HGS: 231–299 mmHg), and low (HGS: 0–230 mmHg) [35].

### 2.7. Diet Information and Physical Activity

During the study period, all participants were instructed not to change their normal eating habits and record accurately the amount, type of food, and fluid consumed using food diaries. Then, dietary information for each participant were extensively referred according to reference dietary intakes for physically active people [36]. In addition, physical activity for each subject was evaluated during 7 consecutive days by using ACTi graph GT1M accelerometer (model WAM 7164; Fort Walton Beach, FL). The average intensity of PA was calculated from the total number of minutes each patient participated in sport activity different intensities. This intensity was based mainly on count thresholds and daily activity counts per minute. Subjects with less accelerometer counts (≤100 counts/min) were characterized by a sedentary lifestyle [37, 38].

According to energy expenditure, PA of all participants was classified into low or sedentary activity (thresholds are less than 4 metabolic equivalents (METs)), moderate activity (thresholds of 4 metabolic equivalents (METs)), and vigorous activity (thresholds of 7 METs), respectively, as previously mentioned, whereas 1 MET refers to either energy expenditure of 1 kcal/kg/h or oxygen uptake in 3.5 mL/kg/min during a quiet sitting position [39].

### 2.8. Assessment of 25-Hydroxyvitamin D and PTH

From freshly separated serum samples of each patient, serum vitamin 25(OH)D level and intact PTH concentrations were estimated as previously reported [25, 26, 28]. A direct competitive chemiluminescence immunoassay with a Liaison auto-analyzer (Liaison, DiaSorin, Turin, Italy) was used to estimate both total 25-hydroxyvitamin (25(OH)D3) and intact PTH in serum of all participants. Based on the manufacturer's instruction, serum concentrations of <10 ng/mL 25(OH)D3 were defined as severe VitD deficiency and <30 ng/mL 25(OH)D3 as VitD insufficiency, whereas a range of 30-100 ng/mL 25(OH)D3 was considered normal.

### 2.9. Liver Histopathology

For all CHC patients to establish the diagnosis and the stage of liver injury, all patients underwent a percutaneous liver biopsy. An automatic 16-gauge tru-cut needle (biopsy gun) which provides adequate specimens for evaluation and fewer cases with tissue fragmentations was used for taking liver biopsies as previously mentioned [16, 40]. For better histological evaluation, the analyzed specimens of liver biopsy should at least 15–25 mm long with complete portal tracts (10 CPTs) [40]. Formalin-fixed, paraffin-embedded sections were stained with hematoxylin and eosin and with Masson's Trichrome. Slides were labeled with patient identification numbers and then reviewed and graded blindly by a senior pathologist; the mean length of liver biopsy and the number of portal tracts were assessed (including only the complete, intact portal tracts) [40]. The degree of fibrosis was scored according to the METAVIR system, and no fibrosis was defined as F0, mild fibrosis as F1, moderate fibrosis as F2, severe fibrosis as F3, and cirrhosis as F4. Significant fibrosis was also defined as F2–F4. Hepatic inflammatory activity was also scored [40].

### 2.10. Statistical Analysis

The statistical power calculations for the selected sample size of 160 subjects were shown to identify a power of 98% and a significance level of 0.05 with an expected frequency of 8.5%.

The results obtained were expressed as the mean and standard deviation. Among groups, Kruskal–Wallis one-way ANOVA and post hoc (Tukey HSD) test were used to compare the mean values of the studied variables. Additionally, Spearman's rank correlation analysis was performed to assess the relationship between various study parameters. The predictive values of vitamin D status; liver fibrosis markers (fibronectin (FN), hydroxyproline (Hyp), and hyaluronic acid (HA)) and their respective indices (HA index, Hyp index, and FN index); and the current clinical malnutrition assessment tools (SGA, BMI, MAMC, and HGS) were examined using stepwise linear regression analysis. Variables that have the highest *R*-squared and strong significance were added in this model. Only, in this study, FN, HA, HAI, HypI, FNI, APRI, vitamin D, Ca and vitamin D intake, SGA, BMI, MAMC, and HGS showed higher *R*-squared and strong significance, whereas PA, basal metabolic rate (BMR kcal/day), total energy expenditure (TEE, kcal/day), and sun exposure showed lower *R*-squared and were deleted from the proposed model. A statistical software SPSS version 18 was used, and the data obtained were deemed significant at *p* < 0.05.

## 3. Results

A total of 80 CHC patients were included in this study (Table 1). The mean age of the patients was 48.2 ± 4.5, and most of them are male (75%). Based on molecular and genotype analysis, the most frequently detected genotype was 4 (77.5%) along with 22.5% of patients with mixed HCV genotypes 2 and 4. Positive HCV patients showed HCV overloads of 48.9 ± 11.3 with higher expression of HCV-RNA (IU/mL) (12.8 × 10^5^). The significant fibrosis was found in 92.3% of CHC patients using the METAVIR system. Most of patients had grade A2–A3 inflammation (68.7%), while only a minority had grade A0–A1 with 31.3%.

On the other hand, liver biopsies from the majority of patients had stage F0–F1 (22.5%) of mild fibrosis and stage (F2–4) (77.5%) of significant fibrosis, while only about 31.3% had cirrhosis (F4) (Table 1). Also, patients with CHC virus showed lower values of adiposity markers BMI and WHtR compared to healthy controls.

Laboratory confirming biomarkers were also estimated in CHC patients and control subjects. CHC patients showed a significant increase in the levels of AST, ALT, TNF-*α*, AFP, and bilirubin and a decline in the values of albumin and platelet counts compared to healthy control subjects as shown in Table 1. Similarly, a decrease in serum vitamin 25(OH)D (ng/mL) levels and an increase in PTH (pg/mL) levels were significantly reported in CHC patients compared to control subjects.

In addition, using SGA scores, malnutrition was estimated among CHC patients (Table 1). Only 15% of the patients showed normal nutrition (SGA score A), and 85% of the study population had moderate to severe SGA scores (B and C) of malnutrition; they are classified into 31.3% with moderately malnourished SGA score (B) and 53.7% with severe malnourished SGA score (C). In CHC patients, malnutrition was shown to be significantly (*p* = 0.001) linked with poor PA levels, daily sunexposure, diet scores, and lower adminstration of both vitamin D and Ca intakes.

In this study, muscle mass and strength were estimated as measures of functional capacity associated with malnutrition in CHC patients (Table 1). Thus, HGS of both arms and MAC, TSF, and MAMC were measured in all CHC patients and control subjects. Compared to control subjects, lower significant values of HGS, MAC, TSF, and MAMC were reported in CHC patients. The data confirmed significant functional loss in muscle mass and physical performance in CHC patients compared to control subjects (Table 1). This may be attributed to malnutrition, lower vitamin D, and Ca intake which leads to reduction in body cell mass.

Based on assessed malnutrition scores (SGA scores), a significant increase in the levels of AST, ALT, TNF-*α*, AFP, bilirubin, and INR and a decline in the values of BMI, WHtR, albumin, and platelet counts were reported in CHC patients with moderate (B) and severe (C) SGA scores compared to those who had normal diets (Table 2).

**Figure 1:**
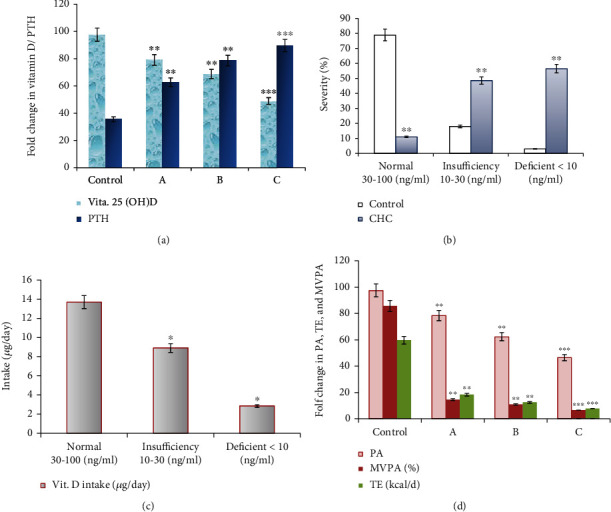
Serum vitamin 25(OH)D and PTH levels, vitamin D deficiency score, related vitamin D intake, and physical activity (PA) scores for healthy controls and CHC patients with different nutritional assessment scores; SGA scores (a–c). (a) Significant difference in serum vitamin 25(OH)D and PTH levels in CHC patients with A (*p* = 0.01), B (*p* = 0.01), and C (*p* = 0.001) nutritional SGA scores compared to healthy controls. (b) Severity of vitamin 25(OH) deficiency among control and CHC patients. CHC patients showed higher ratios of vitamin 25(OH)D insufficiency and deficiency (*p* = 0.01) compared to control subjects. (c) Lower vitamin D intake was significantly reported in CHC patients with both insufficiency and deficiency in vitamin 25(OH)D levels (*p* = 0.05). (d) PA scores, TE (kcal/d), and MVPA (%) were significantly reduced in CHC patients with A (*p* = 0.01), B (*p* = 0.01), and C (*p* = 0.001) nutritional SGA scores compared to healthy controls. PTH: parathyroid hormone; MVPA: moderate to vigorous physical activity; PA: physical activity; SGA: subjective global assessment; A: well nourished; B: moderately malnourished; C: severely malnourished; TE: total energy (kcal/d).

Also, in moderate and severely malnourished CHC patients who received diets containing insufficient vitamin D and Ca, a significant increase in vitamin 25(OH)D (ng/mL) levels and an increase in the levels of PTH (pg/mL) were significantly reported compared to control subjects and CHC patients who had normal diets with adequate vitamin D and Ca as shown in Table 2 and Figure 1(a). In the same line, vitamin D deficiency was shown to be significantly increased in malnourished CHC patients who received diets with inadequate vitamin D supplements compared to control and CHC with normal diets as in Figures 1(b) and 1(c). Similarly, poor PA levels with lower TE were significantly correlated with inadequate diets and vitamin D in moderate and severely malnourished CHC patients, whereas PA scores, TE (kcal/d), and MVPA (%) were significantly reduced in CHC patients with A (*p* = 0.01), B (*p* = 0.01), and C (*p* = 0.001) nutritional SGA scores compared to healthy controls (Figure 1(d)).

**Figure 2:**
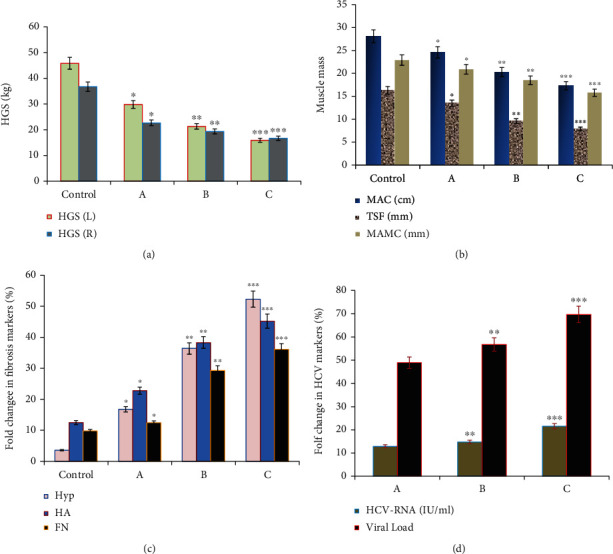
Handgrip strength, muscle mass parameters (MAC, TSF, and MAMC), and related HCV markers and liver fibrosis markers in healthy control and CHC patients based on different nutritional assessment scores; SGA scores (A: well nourished; B: moderately malnourished; C: severely malnourished). (a) HGS scores were significantly reduced in CHC patients with severe (*p* = 0.001) and moderate (*p* = 0.01) malnutrition compared to those of the normal nutrition (*p* = 0.01) and control groups, respectively. (b) Muscle mass indices (MAC, *p* = 0.05; TSF, *p* = 0.01; and MAMC; *p* = 0.001) showed significant reduction in all CHC patients with normal and malnutrition SGA scores compared to control subjects with normal nutrition scores. (c) The levels of HYP, HA, and FN as markers of liver fibrosis were significantly increased in CHC patients with A (*p* = 0.01), B (*p* = 0.01), and C (*p* = 0.001) nutritional SGA scores compared to healthy controls. (d) HCV-RNA (IU/mL) and HCV viral load as related HCV markers were significantly highly expressed in CHC patients with moderate (SAG B score; *p* = 0.01) and severe (SAG C score; *p* = 0.001) malnutrition compared to those who were well nourished (SAG A score). MAC: midarm circumference in cm; TSF: triceps skin fold thickness in mm; MAMC: midarm muscle circumference in mm; CHC patients: chronic hepatitis C patients; HYP: hydroxyproline; HA: hyaluronic acid; FN: fibronectin; SGA: subjective global assessment; A: well nourished; B: moderately malnourished; C: severely malnourished.

Also, HGS scores of both arms, MAC, TSF, and MAMC as outcome measures of functional loss in muscle mass and physical performance were reported to be closely correlated with malnutrition SGA scores in CHC patients. HGS scores were significantly reduced in CHC patients with severe (*p* = 0.001) and moderate (*p* = 0.01) malnutrition compared to those of normal nutrition (*p* = 0.01) and control groups, respectively (Figure 2(a)). In addition, muscle mass indices (MAC, *p* = 0.05; TSF, *p* = 0.01; and MAMC, *p* = 0.001) showed significant reduction in moderate and severely malnourished CHC patients compared to those who had normal diets and control subjects (Figure 2(b)).

In this current study, HYP, HA, and FN as markers of liver fibrosis were estimated in CHC patients. HYP, HA, and FN were significantly increased in CHC patients with A (*p* = 0.01), B (*p* = 0.01), and C (*p* = 0.001) nutritional SGA scores compared to healthy controls (Figure 2(c)). Also, expression of HCV-RNA (IU/mL) and viral overloads significantly increased in moderate and severely malnourished CHC patients compared to those who were well nourished as shown in Figure 2(d).

Regarding the presence of significant fibrosis and cirrhosis, CHC patients with significant cirrhosis (F4) showed significant (*p* = 0.001) increase in the levels of AST, ALT, TNF-*α*, AFP, bilirubin, INR, APRI, Hyp index, HA index, and FN index; a decline in the values of vitamin 25(OH)D (ng/mL); and an increase in the levels of PTH (pg/mL) with reduction in platelet counts compared to CHC patients with mild (F0–F1) and significant fibrosis (F2–F4), respectively (Table 3).

Also, higher functional loss in muscle mass and physical performance was reported in CHC patients with significant cirrhosis (F4). HGS scores of both arms, MAC, TSF, and MAMC were significantly (*p* = 0.001) reduced with lower vitamin D and Ca intake in malnourished CHC patients with significant cirrhosis (F4) compared to CHC patients with mild (F0–F1) and significant (F2–F4) fibrosis, respectively (Table 3).

Malnutrition, adiposity, handgrip strength, muscle mass, and vitamin D and Ca intake were shown to be significantly correlated with the degree of liver fibrosis in CHC patients. The degree of significant fibrosis and cirrhosis correlated positively with adiposity, lower vitamin 25(OH)D, and increased levels of PTH, diet scores, and vitamin D and Ca intake and negatively with SGA malnutrition scores, HGS, and muscle mass indices (MAC, TSF, and MAMC, respectively) (Table 4).

The data also, confirmed that Hyp, HA, FN, APRI, HypI, HAI, and FNI as noninvasive biomarkers could be used separately or in association with vitamin D status and HGS as predictors for both liver fibrosis and malnutrition in CHC patients. Stepwise regression analysis revealed that adiposity, liver fibrosis markers (Hyp, HA, FN, APRI, HypI, HAI, and FNI), muscle mass scores (MAC, MAMC, TSF, HGS, and SGA), and vitamin D deficiency were associated with ~70.5%–89.6% of the incidence of malnutrition in CHC patients with significant fibrosis and cirrhosis (Table 5).

## 4. Discussion

In this study, malnutrition was cross-sectionally surveyed in 80 CHC patients and was shown to be significantly associated with severity of liver fibrosis. Patients with well-nourished status was predicted in 15% of the study population, and 85% of the study population had major changes in nutritional status; they were classified into 31.3% with a moderate malnourished SGA score (B), and 53.7% of the study population had a severe malnourished SGA score (C). In addition, the results revealed that adiposity, liver fibrosis markers (Hyp, HA, FN, APRI, HypI, HAI, and FNI), muscle mass scores (MAC, MAMC, TSF, HGS, and SGA), and vitamin D deficiency were associated significantly with ~70.5%–89.6% of the incidence of malnutrition in CHC patients with significant fibrosis and cirrhosis.

Malnutrition shows variability from 20% in patients with compensated disease to more than 60% in patients with liver cirrhosis, which depends mainly on the type of the method used for evaluation [2–4]. In patients with chronic hepatitis C (CHC), malnutrition was reported early in the course of HCV and proceeds progressively throughout the spectrum of HCV disease [4].

In CHC patients, the rapid progression of liver damage was shown to be associated with malnutrition [5]. Frequently, CHC patients become malnourished whereas they are unable to meet their nutritional requirements [6].

85% of our patients were malnourished (SGA B or C) which was higher than the reported 28% percentage previously estimated in cirrhotic patients [41]. The difference may be partially explained on the basis of the difference in clinical severity of liver fibrosis among our CHC patients. Similar to our SGA prevalence rates, other reported studies showed statistically significant differences in the BMI and overall score of SGA throughout the three times of SGA assessment. According to these studies, chronic hepatitis C study subjects lying in category A were 83.3%, 63.7%, and 75.5% in the 1st, 2nd, and 3rd SGA assessment trials, respectively, and the rest of CHC patients fall in category B. Their data showed that nutritional assessment is authoritative for better clinical outcome in patients with chronic hepatitis C treated with combination therapy [42].

In addition, in this study, significant correlation was reported between estimated SGA scores and severity of liver fibrosis as measured by increased liver dysfunction, AST, ALT, TNF-*α*, AFP, bilirubin, INR; a decline in the values of BMI, WHtR, and albumin; and a significant increase in the levels of Hyp, HA, and FN and their respective indices (APRI, Hyp, HAI, and FNI) in CHC patients with significant fibrosis and cirrhosis. In previous studies, a similar correlation was significantly (*p* = 005) reported between SGA and prognosis of cirrhosis as measured by Child-Pugh scores [30]. Recently, it was reported that malnutrition was significantly associated with severity of liver fibrosis and that nutritional SGA scores could be used to predict liver severity and short-term survival in cirrhotic patients [24].

Vitamin D is the best well-known naturally regulators for both bone mineralization and calcium homeostasis [43]. In addition, it has many other pivotal physiological roles in living cells, such as cellular proliferation, apoptosis, differentiation, and inflammation [43].

In patients with HCV infection, vitamin D status was shown to be inversely correlated with liver fibrosis progression, liver dysfunction, and cellular damage in treated and nontreated naive CHC stages [44]. Lower serum vitamin D levels have been reported in patients with chronic liver disease from different etiologies [45]. The deficiency in vitamin D levels was associated with the pathogenesis of liver cells in 46% to 92% of CHC patients [45].

In this study, a reduction in serum level of vitamin 25(OH)D and an increase in PTH levels were significantly reported in CHC patients compared to control subjects. Deficiency of vitamin 25(OH)D and increased levels of PTH were significantly correlated to liver severity. Significant reduction in the levels of vitamin 25(OH)D and increased levels of PTH were significantly reported in cirrhotic CHC cases compared to those with significant fibrosis.

Previously, it was reported that adequate vitamin D levels obtained through normal diets or by supplements significantly affect HCV replication in liver cells via immunomodulatory effect [10, 11, 44, 45]. Also, vitamin D had a direct inhibitory effect on viral production by regulating the expression of interferon-beta (INF-*β*) and consequent increase of innate immune response of infected liver cell against further HCV propagation. Based on this study, deficiency in vitamin D among our CHC patients results in a reduction in immunity which gives a good universe for more expression and prognosis of HCV, resulting in severe liver fibrosis [44, 45].

In addition, in our CHC patients with cirrhosis, an increase in the levels of AFP and TNF-*α* as inflammatory cytokines was recorded; this may be due to the fact that vitamin D deficiency produces an increase in intrahepatic inflammation, circulating levels of several inflammatory cytokines, and chemokines closely related to disease progression as previously reported [44–46].

Biological activity of vitamin D in normal cells were performed in combination with several other hormones, including parathyroid hormone (PTH). During vitamin D deficiency, levels of ionized calcium were declined [47]. This in turn activates the increase of PTH levels to compensate loss of calcium via stimulation of bone resorption to the release of calcium. Thus, PTH level is often used as an index of vitamin D repletion whereas adequacy of vitamin D levels is obtained in normal cells when PTH levels are maximally suppressed [46–49].

Previously, vitamin D deficiency in HCV-infected patients was inversely correlated with elevated PTH serum levels. In normal control subjects with vitamin D values above approximately 30 ng/mL, serum PTH levels were shown to be at low steady levels [1–11].

The changes in the levels of vitamin 25(OH)D and PTH levels were significantly correlated with malnutrition in CHC patients. In this study, CHC patients with moderate and severe malnutrition ASG score showed a significant decline in vitamin D and an increase in PTH levels, especially in CHC patients who received inadequate amounts of vitamin D and Ca in their foods.

The data obtained were in line with those who reported that the incidence of liver diseases significantly influences physiological process relating to nutrient digestion, absorption, storage, and metabolism. These collectively may lead to deficiency in vitamin, mineral, and protein-energy malnutrition [2, 4, 5, 12]. Previous research studies showed that hepatitis C patients with or without liver complications are already at risk of malnutrition and inadequate dietary intake and that nutrient deficiencies especially those of vitamin D and Ca have a direct impact on the clinical history of patients with liver disease [7, 33].

Thus, diagnosing and treating malnutrition associated with HCV caused before the onset of complications particularly cirrhosis and hepatocellular carcinoma are significantly needed for new methods for assessing nutritional status [7, 29, 33].

In this study, HGS scores of both arms, MAC, TSF, and MAMC as measures of loss in muscle mass and functional physical performance were significantly correlated with malnutrition in CHC patients, especially those with severe liver disease. A significant decrease in HGS scores and muscle mass indices (MAC, TSF, and MAMC) were observed in severely malnourished CHC patients with significant fibrosis and cirrhosis; however, more reduction in HGS scores and muscle mass indices was observed in cirrhotic patients (F4) compared to those with significant fibrosis (F2–F3).

According to the HGS, previous studies found that a significant risk of malnutrition was reported in CHC patients. In addition to that, they concluded that HGS could provide a good alternative method in assessing a decline in protein and to be a superior indicator in detecting cirrhotic patients with malnutrition [7, 30, 33], whereas protein depletion significantly affects HGS [84–85]. In early stages of cirrhosis, the influence on muscle strength was significantly well measured by HGS than evaluation by of MAC, TSF, and MAMC which suffer from low changes [85]. In the same manner, a significant change in HGS scores was reported compared to those of MAC, TSF, and MAMC in studied CHC patients [29, 31–36].

In this study, severity of liver disease correlated positively with adiposity, diets containing in adequate amounts of vitamin D and Ca intake, vitamin 25(OH)D deficiency, and increased levels of PTH and negatively with SGA malnutrition scores, HGS, and muscle mass indices (MAC, TSF, and MAMC, respectively). Past studies use different parameters such as body mass index (BMI) and subjective global assessment (SGA) along with the handgrip strength (HGS) for the evaluation of malnutrition associated with liver cirrhosis [36–40].

Although liver fibronectin, hydroxyproline, hyaluronic acid, liver function, and platelet counts and their respective indices (APRI, HA index, Hyp index, and FN index) as noninvasive biomarkers were reported in evaluation of liver fibrosis [25, 26, 28, 34, 35], little is known about the importance of parameters as noninvasive biomarkers in predicating and evaluation of malnutrition associated with severe hepatic dysfunction.

Thus, in this study, Hyp, HA, FN, APRI, HypI, HAI, and FNI as noninvasive biomarkers could be used separately or in association with vitamin D status and HGS as predictors for both liver fibrosis and malnutrition in CHC patients. Data of stepwise regression analysis revealed that adiposity, liver fibrosis (Hyp, HA, FN, APRI, HypI, HAI, and FNI), muscle mass scores (MAC, MAMC, TSF, HGS, and SGA), and vitamin D deficiency were associated with ~70.5%–89.6% of the incidence of malnutrition in CHC patients with significant fibrosis and cirrhosis.

Previously, compared to BMI and muscle mass indices, only HGS and SGA could predict severity and short survival rates in early and late stages of cirrhotic livers [30, 36–49]. In this study, vitamin D deficiency, increased PTH, and noninvasive liver biomarkers (Hyp, HA, FN, APRI, HypI, HAI, and FNI) could be a new trend of markers measuring malnutrition associated with liver severity in CHC-patients.

## 5. Conclusions

The results showed that deficiency in vitamin D levels, HGS, SGA, and muscle mass scores (MAC, MAMC, or TSF) could be used as markers of liver pathogenicity in patients with CHC. In addition, the study concluded that noninvasive biomarkers Hyp, HA, FN, APRI, HypI, HAI, and FNI separately or in association with vitamin D status, HGS, SGA, and muscle mass scores (MAC, MAMC, or TSF) were significantly associated with an incidence of malnutrition between~70.5% and 89.6% of CHC patients with significant fibrosis and cirrhosis.

## Figures and Tables

**Table 1:** Demographic data, laboratory, physical activity, diet scores, and histological characteristics of chronic hepatitis C patients and healthy control subjects.

Characteristics	All CHC patients, *N*, mean ± SD (%)	Healthy controls, *N*, mean ± SD (%)	*p* value
No.	80	80	—
Age (year)	48.2 ± 4.5	47.8 ± 4.5	0.11
Gender (male/female)	60/20	60/20	0.18
Anthropometry			0.001
BMI	21.8 ± 3.2	24.6 ± 4.7	
WHtR	0.42 ± 0.09	0.75 ± 0.13	
Muscle mass			0.001
MAC (in cm)	23.3 ± 2.6	28.1 ± 4.7	
TSF (in mm)	12.8 ± 6.4	16.3 ± 3.1	
MAMC (in mm)	18.9 ± 5.7	22.9 ± 4.2	
Diet measurements			0.001
Diet score	11.3 ± 3.8	28.5 ± 4.9	
Dietary vitamin D intake (IU/d)	86.8 ± 48	215 ± 96	
Dietary Ca intake (mg/d)	650 ± 89	1400 ± 185	
SGA			0.001
A (well nourished)	12 (15%)	76 (95%)	
B (moderately malnourished)	25 (31.3%)	4 (5%)	
C (severely malnourished)	43 (53.7%)	0 (0%)	
PA			0.001
Total PA (counts/min)	398 ± 75	3850 ± 480	
MVPA (%)	14.6	85.9	
Total energy (kcal/d)	1180 ± 318	6450 ± 518	
Sun exposure (h/day)	1.4 ± 0.6	5.4 ± 2.8	0.001
Handgrip (kg)			0.001
Right hand	18.9 ± 4.8	41.9 ± 4.8	
Left hand	22.6 ± 3.7	29.6 ± 3.7	
AST (IU/mL)	68.4 ± 24.7	19.6 ± 5.6	0.001
ALT (IU/mL)	85.3 ± 42.8	23.5 ± 4.3	0.001
Albumin	2.9 ± 0.49	4.5 ± 1.8	0.001
Bilirubin	8.65 ± 6.3	0.85 ± 0.29	0.001
TNF-*α* (pg/mL)	34.7 ± 6.8	11.2 ± 3.2	0.001
AFP	28.3 ± 6.3	4.6 ± 1.9	0.001
Vitamin 25(OH)D (ng/mL)	14.7 ± 3.2	45.6 ± 8.2	0.001
PTH (pg/mL)	56.2 ± 4.1	13.7 ± 2.8	0.001
Platelets (109/L)	189 ± 36.9	315 ± 51.7	0.001
Duration of HCV (years)	7.6 ± 3.4	—	—
HCV-RNA (IU/mL)	12.8 × 10^5^	—	—
HCV genotypes			—
G4	62 (77.5%)	—	
G2 and 4	18 (22%)	—	
Viral load	48.9 ± 11.3	—	—
Stage of fibrosis (METAVIR), *n* (%)	74/80 (92.3%)	—	—
F0	6 (7.5%)		
F1	12 (15%)		
F2	15 (18.75%)		
F3	22 (27.5%)		
F4	25 (31.3%)		
Population, *n*		—	—
F0-F1	18 (22.5%)		
F2-F4	62 (77.5%)		
F0-F3	55 (68.75%)		
F4	25 (31.3%)		
Mean length of liver biopsy core (LBC + SD)	18.9 ± 0.89 cm	—	—
Mean number of portal tracts (NoP + SD)	15 ± 3.8	—	—
Necroinflammation		—	—
A0–A1	25 (31.3%)		
A2–A3	55 (68.7%)		

All values were reported as the mean ± SD or median (interquartile range) or percentage. Kruskal–Wallis one-way ANOVA and post hoc (Tukey HSD) test were used to compare the mean values of the studied variables. Variables were considered significantly different at *P* < 0.05. BMI: body mass index; WHtR: waist to height ratio; MVPA: moderate to vigorous physical activity; PA: physical activity; HGS: handgrip strength; SGA: subjective global assessment; AFP: *α*-Fetoprotein; TNF-*α*: tumor necrosis factor-alpha; MAC: midarm circumference in cm; TSF: triceps skin fold thickness in mm; MAMC: midarm muscle circumference in mm.

**Table 2:** Comparison of adiposity, diets, and other laboratory biomarkers in CHC patients based on nutritional assessment scores (SGA scores).

Variables	SGA classes (mean ± SD)			*p* value
	A (*n* = 12)	B (*n* = 25)	C (*n* = 43)	
Anthropometry				0.001
BMI	21.2 ± 3.9	18.1 ± 2.8	16.9 ± 2.5	
WHtR	0.69 ± 0.12	0.49 ± 0.84	0.29 ± 0.68	
Diet measurements				0.001
Diet score	10.9 ± 3.6	8.2 ± 3.2	6.3 ± 2.1	
Dietary vitamin D intake (IU/d)	89.5 ± 51	72.8 ± 48	65.8 ± 32	
Dietary Ca intake (mg/d)	696 ± 94	636 ± 41	548 ± 26	
AST (IU/mL)	62.3 ± 21.5	68.1 ± 21.9	76.3 ± 23.4	0.001
ALT (IU/mL)	79.8 ± 31.2	82.4 ± 28.3	92.7 ± 28.7	0.001
Albumin	3.7 ± 0.6	2.97 ± 0.49	2.48 ± 0.31	0.001
Bilirubin	2.86 ± 5.9	5.8 ± 2.6	8.96 ± 6.3	0.001
TNF-*α* (pg/mL)	33.9 ± 4.8	38.2 ± 5.1	48.7 ± 6.3	0.001
AFP	29.8 ± 5.3	32.2 ± 3.7	41.9 ± 6.8	0.001
INR	1.25 ± 0.18	1.8 ± 1.1	2.5 ± 1.18	0.001
Platelets (109/L)	192 ± 32.7	178 ± 18.3	96 ± 16.7	0.001

All values were reported as the mean ± SD or median (interquartile range) or percentage. Kruskal–Wallis one-way ANOVA and post hoc (Tukey HSD) test were used to compare the mean values of the studied variables. Variables were considered significantly different at *P* < 0.05. BMI: body mass index; WHtR: waist to height ratio; SGA: subjective global assessment; AFP: *α*-Fetoprotein; TNF-*α*: tumor necrosis factor-alpha; INR: international normalized ratio of prothrombin.

**Table 3:** Association of malnutrition, vitamin D deficiency, handgrip strength, muscle mass, and other related liver fibrosis markers with the presence of significant fibrosis and cirrhosis in CHC.

Variables	Significant fibrosis (mean ± SD)		*p* value	Cirrhosis (mean ± SD)		*p* value
	F0–F1	F2–F4		F0–F3	F4	
AST (IU/mL)	65.7 ± 11.3	81.7 ± 16.7	0.001	78.3 ± 12.4	125.3 ± 18.4	0.001
ALT (IU/mL)	72.7 ± 6.8	112 ± 11.8	0.001	100 ± 9.8	186 ± 21.3	0.001
INR	1.2 ± 0.14	1.3 ± 1.1	0.001	2.6 ± 0.61	3.7 ± 1.8	0.001
Platelets (109/L)	228.6 ± 21.3	210.8 ± 14.2	0.001	198.6 ± 11.7	161.2 ± 31.8	0.001
HYP (lg/mL)	2.9 ± 1.9	8.6 ± 3.65	0.001	15.2 ± 4.9	18.2 ± 3.9	0.001
HA (ng/mL)	49.5 ± 10.2	136.0 ± 31.2	0.001	186.5 ± 38.4	215.7 ± 48.7	0.001
FN (ng/mL)	16.5 ± 3.6	31.5 ± 3.6	0.001	61.8 ± 12.8	86.9 ± 21.3	0.001
APRI	0.8 ± 0.45	2.6 ± 0.9	0.001	2.85 ± 086	3.6 ± 0.51	0.001
Hyp index	1.96 ± 0.42	5.81 ± 1.2	0.001	8.96 ± 1.6	12.9 ± 3.31	0.001
FNPRI index	2.8 ± 0.68	7.9 ± 2.7	0.001	9.6 ± 1.6	14.9 ± 6.1	0.001
HAPRI	0.86 ± 0.56	1.68 ± 1.3	0.001	1.8 ± 0.86	4.9 ± 1.69	0.001
Vitamin 25 (OH)D (ng/mL)	21.7 ± 2.9	12.3 ± 3.9	0.001	15.2 ± 1.5	9.8 ± 2.3	0.001
PTH (pg/mL)	48.5 ± 3.1	62.2 ± 6.3	0.001	86.7 ± 2.6	96.7 ± 1.9	0.001
Dietary vitamin D intake (IU/d)	96.4 ± 48	81.9 ± 31	0.001	56.8 ± 31	46.7 ± 25	0.001
Dietary Ca intake (mg/d)	615 ± 68	549 ± 74.5	0.001	510 ± 31.6	396 ± 36.1	0.001
Diet score	12.8 ± 1.9	10.3 ± 2.8	0.001	9.6 ± 3.4	6.1 ± 4.7	0.001
SGA						
A (well nourished)	6	12	0.001	15	0	0.001
B (moderately malnourished)	7	23		16	11	
C (severely malnourished)	5	27		24	14	
Handgrip (kg)						
Right hand	21.6 ± 5.3	18.1 ± 3.6	0.001	17.8 ± 3.7	16.8 ± 1.6	0.001
Left hand	23.1 ± 3.7	16.5 ± 2.1		15.8 ± 2.4	14.2 ± 3.8	
Muscle mass scores						
MAC (in cm)	21.5 ± 1.8	19.7 ± 3.6	0.001	17.9 ± 3.1	16.9 ± 1.6	0.001
TSF (in mm)	13.6 ± 5.2	12.1 ± 3.8		11.6 ± 3.8	9.8 ± 4.6	
MAMC (in mm)	17.6 ± 3.7	16.9 ± 2.3		15.9 ± 2.8	14.9 ± 6.3	

SD: standard deviation; APRI: AST to platelet ratio index; Hyp index: hydroxyproline to platelet ratio index; HAPRI: HA to platelet ratio index; FNPRI: FN to platelet ratio index; ALT: alanine aminotransferase; AST: aspartate aminotransferase; PTH: parathyroid hormone; MAC: midarm circumference in cm; TSF: triceps skin fold thickness in mm; MAMC: midarm muscle circumference in mm; CHC patients: chronic hepatitis C patients; HYP: hydroxyproline; HA: hyaluronic acid; FN: fibronectin; SGA: subjective global assessment; A: well nourished; B: moderately malnourished; C: severely malnourished; INR: international normalized ratio of prothrombin. Student's *t*-test was used followed by Mann–Whitney *U* test. *P* values at <0.05 are considered statistically significant.

**Table 4:** Correlations between BMI, muscle mass scores (MAC, MAMC, and TSF), HGS, SGA malnutrition scores, and vitamin D deficiency with fibrosis markers in CHC patients with significant fibrosis and cirrhosis.

Variables	Significant fibrosis (mean ± SD)				Cirrhosis (mean ± SD)			
	F0–F1		F2–F4		F0–F3		F4	
	*r*	*p*	*r*	*p*	*r*	*p*	*r*	*p*
BMI	0.058	0.12	0.12	0.16	0.075	0.001	0.43	0.001
WHtR	0.075	0.412	0.145	0.124	0.168	0.001	0.815	0.001
Vitamin 25(OH)D (ng/mL)	0.315	0.01	0.512	0.01	0.365	0.001	0.618	0.001
PTH (pg/mL)	0.115	0.01	0.258	0.01	0.519	0.001	0.618	0.001
Dietary vitamin D intake (IU/d)	0.145	0.01	0.235	0.01	0.415	0.001	0.357	0.001
Dietary Ca intake (mg/d)	0.125	0.01	0.321	0.01	0.632	0.001	0.617	0.001
Diet score	0.112	0.01	0.226	0.01	0.418	0.001	0.628	0.001
SAG score	-0.365	0.01	-0.253	0.01	-0.452	0.001	-0.315	0.002
Handgrip (kg)	-0.325	0.001	0.416	0.001	-0.489	0.001	-0.257	0.001
MAC	-0.089	0.001	0.164	0.001	-0.265	0.001	-0.147	0.001
TSF	-0.032	0.01	0.187	0.01	-0.163	0.01	-0.129	0.01
MAMC	-0.096	0.001	0.265	0.001	-0.348	0.001	-0.318	0.001

PTH: parathyroid hormone; MAC: midarm circumference in cm; TSF: triceps skin fold thickness in mm; MAMC: midarm muscle circumference in mm; CHC patients: chronic hepatitis C patients; SGA: subjective global assessment. Pearson's (*r*) coefficient and *P* values at <0.05 are considered statistically significant.

**Table 5:** Stepwise multiple regression analysis for malnutrition predicted by adiposity, liver fibrosis, muscle mass scores (MAC, MAMC, and TSF), HGS, SGA malnutrition scores, and vitamin D deficiency in CHC patients with significant fibrosis and cirrhosis.

Variables	Significant fibrosis (F_2_-F_3_)		Significant cirrhosis (F_4_)	
	*R*2(*β*)^∗^	95% CI	*R*2(*β*)^∗∗^	95% CI
Adiposity (BMI)	4.6 (0.35)	89 (75–100)	5.9 (0.38)	86 (75–98)
Vitamin D deficiency	23.8 (0.42)	92 (82–98)	16.9 (0.62)	81 (68–96)
Ca and vitamin D intake	5.6 (0.53)	75 (65–89)	6.3 (0.47)	90 (88–100)
MAC	2.8 (0.28)	84 (72–92)	4.3 (0.32)	94 (88–100)
MAMC	3.6 (0.22)	65 (55–90)	5.3 (0.32)	78 (88–100)
TSF	1.6 (0.29)	76 (65–89)	4.6 (0.37)	84 (88–100)
SGA	3.7 (0.33)	79 (68–92)	5.8 (0.48)	91 (82–100)
HGS	4.1 (0.53)	89 (65–89)	6.8 (0.51)	96 (88–100)
HYP (lg/mL)	2.6 (0.33)	91 (80–100)	3.9 (0.25)	97 (88–100)
HA (ng/mL)	3.6 (0.31)	95 (80–100)	4.9 (0.31)	89 (88–100)
FN (ng/mL)	1.6 (0.21)	91 (86–100)	3.9 (0.38)	95 (88–100)
APRI	2.6 (0.35)	98 (86–100)	4.5 (0.41)	98 (85–100)
Hyp index	4.2 (0.41)	87 (76–98)	5.9 (0.46)	90 (85–100)
FNPRI index	3.2 (0.52)	89 (72–96)	4.9 (0.39)	89 (82–100)
HAPRI	2.9 (0.61)	93 (76–96)	5.6 (0.68)	96 (82–100)
*ΣR*2 (%)	70.5 (0.38)	96 (76–96)	89.6 (0.39)	98 (88–100)

^∗^ *p* < 0.01 and ^∗∗^*p* < 0.001. *ΣR*2 = summation of cumulative values of *R* relating to studied variables. CI: confidence interval; BMI: body mass index; MAC: midarm circumference in cm; TSF: triceps skin fold thickness in mm; MAMC: midarm muscle circumference in mm; CHC patients: chronic hepatitis C patients; SGA: subjective global assessment; HYP: hydroxyproline; HA: hyaluronic acid; FN: fibronectin; APRI: AST to platelet ratio index; Hyp index: hydroxyproline to platelet ratio index; HAPRI: HA to platelet ratio index; FNPRI: FN to platelet ratio index.

## Data Availability

All data generated or analyzed during this study are presented in the manuscript. Please contact the corresponding author for access to data presented in this study.
